# Are Manipulation Checks Necessary?

**DOI:** 10.3389/fpsyg.2018.00998

**Published:** 2018-06-21

**Authors:** David J. Hauser, Phoebe C. Ellsworth, Richard Gonzalez

**Affiliations:** ^1^Department of Psychology, University of Southern California, Los Angeles, CA, United States; ^2^University of Michigan, Ann Arbor, MI, United States

**Keywords:** manipulation checks, experimental methods, research design, mediation, emotion

## Abstract

Researchers are concerned about whether manipulations have the intended effects. Many journals and reviewers view manipulation checks favorably, and they are widely reported in prestigious journals. However, the prototypical manipulation check is a verbal (rather than behavioral) measure that always appears at the same point in the procedure (rather than its order being varied to assess order effects). Embedding such manipulation checks within an experiment comes with problems. While we conceptualize manipulation checks as measures, they can also act as interventions which initiate new processes that would otherwise not occur. The default assumption that manipulation checks do not affect experimental conclusions is unwarranted. They may amplify, undo, or interact with the effects of a manipulation. Further, the use of manipulation checks in mediational analyses does not rule out confounding variables, as any unmeasured variables that correlate with the manipulation check may still drive the relationship. Alternatives such as non-verbal and behavioral measures as manipulation checks and pilot testing are less problematic. Reviewers should view manipulation checks more critically, and authors should explore alternative methods to ensure the effectiveness of manipulations.

## Introduction

The past decade has witnessed a growing malaise about the validity of scientific research, particularly in medicine and psychology (Ioannidis, 2005; Pashler and Wagenmakers, 2012; Open Science Collaboration, 2015). Almost all of this concern focuses on decisions that the researcher makes after the study has already been designed and run – decisions about which data and which analyses to include in the final report and how to describe them. The only pre-analysis issues that have received much attention are the preregistration of hypotheses and the choice of an appropriate *n*.

Relatively little attention has been paid to the actual design and procedure of the study – for example, to the choice of control groups or the design of the independent and dependent variables – and these methodological choices are at least as important contributors to the validity of research. If the research has a confound, or the measures are inappropriate, or the participants are inattentive, the results will be inconclusive or misleading (Campbell and Stanley, 1963). The study may have a perfect replication record but still be meaningless. This article is about the use of manipulation checks, and by extension, any other obtrusive measures that occur between the treatment and the dependent variable measure. Concerns about manipulation checks are not novel (e.g., Sigall and Mills, 1998); the problem is not that the best practices are unknown, but that they are often neglected.

Are manipulation checks necessary? A common view is that every experimental study benefits by the inclusion of a manipulation check and suffers when it fails to include one. A recent survey of social psychologists at an international meeting found that more than 75% believed that a manipulation check is “necessary in a well-designed social psychology lab experiment” (Fayant et al., 2017). Reviewers can regard the failure to include a manipulation check as a serious flaw, tantamount to non-random assignment or lack of an appropriate control group, and can regard it as more serious than using experimenters who are aware of the participants’ condition or using participants’ reports of what they think they would do as evidence of what they would actually do. The registered replication report format (now housed in *Advances in Methods and Practices in Psychological Sciences*) requires proposers to implement and reviewers to identify any “necessary manipulation checks” (Simons and Holcombe, 2014).

Just how common are manipulation checks in psychological research? We cataloged every empirical article appearing in *JPSP:ASC, Psychological Science, JEP:Gen, PSPS*, and *JESP* in 2015 and the first 2 months of 2016. For each article, we coded whether a manipulation check was used and features of the manipulation check that could make it potentially problematic: if it was a purely verbal (as opposed to behavioral) measure and if it always occurred at the same point in the experimental procedure (rather than varying the order so that potential order effects could be assessed).

As shown in **Table 1**, manipulation checks were common, appearing in a third (33%) of the articles sampled. They are considerably more common in social psychology journals (JPSP:ASC – 63%, JESP – 53%, PSPB – 41%) than in general psychology journals (JEP:Gen – 22%, PS – 15%). Even more striking was the prevalence of manipulation checks that were potentially problematic. Of the 204 articles that had a manipulation check, the vast majority (*n* = 180; 88%) had a verbal manipulation check and did not manipulate the order in which it appeared. In terms of overall frequency, 29% of articles utilized one of these manipulation checks. Again, this practice was more prevalent in social psychology journals (JPSP:ASC – 54%, JESP – 51%, PSPB – 37%) than in general psychology journals (JEP:Gen – 17%, PS – 11%). Thus, while manipulation checks are common and often encouraged, the best practices for employing them are often neglected.

**Table 1:** Prevalence of manipulation checks of different types in various psychology journals from January 2015 to February 2016.

	JEP: Gen	PS	JPSP: ASC	PSPB	JESP	Total
Number of empirical papers	110	194	35	140	135	614
Number (%) with at least one manipulation check	24 (22%)	29 (15%)	22 (63%)	58 (41%)	71 (53%)	204 (33%)
Number (%) that had a verbal manipulation check	21 (19%)	24 (12%)	19 (54%)	55 (39%)	69 (51%)	188 (31%)
Number (%) that had a verbal manipulation check and did not manipulate question order	19 (17%)	21 (11%)	19 (54%)	52 (37%)	69 (51%)	180 (29%)

## Purposes of Manipulation Checks

The idea of manipulation checks is not new. In one of the first articles about laboratory experimentation as a research method, Festinger warned that “It is rarely safe to assume beforehand that the operations used to manipulate variables will be successful and will tie in directly with the concept the experimenter has in mind. It is a worthwhile precaution to check on the success of the experimental manipulations” (Festinger, 1953, p. 145). Over the years the use of manipulation checks has become more common, and they are used as checks of attention, checks of the effectiveness of the independent variable induction, and checks on mediational processes.

### Checks on Attention

Instead of having to respond to an involving situation, as in the days of the high-impact experiment (Aronson, 2010), participants nowadays are more likely to be sitting alone at a computer pressing keys in response to written stimuli (Baumeister et al., 2007). This raises a concern that may make a manipulation check seem necessary: Did the participant notice the treatment at all? Online materials are typically relatively uninvolving, and the participants may be rushing through the study without paying much attention. If the treatment is a small but crucial change in the wording of a question, its effectiveness requires that the participant actually read the whole question.

A proposed solution to this problem is a new kind of manipulation check, the Instructional Manipulation Check (IMC; Oppenheimer et al., 2009), also called an attention check, a screener, or a trap question. These questions are designed to check on whether participants are actually reading the questions or just skipping to the answer choices. For example, a question may appear to ask what sports a participant plays but include a long paragraph of explanation that contains a brief instruction to ignore the sports answer choices and click on something else. Oppenheimer et al. (2009) found that over 30% of their participants failed this IMC, simply checking one or more of the sports. They also found that only those who passed the IMC were affected by the experimental treatment, suggesting that those who failed to read the IMC carefully also failed to read the question containing the actual manipulation carefully. They recommended using IMCs to eliminate inattentive participants from the analysis and thereby increase the statistical power of the data (for a review of the validity of attention checks, see Thomas and Clifford, 2017).

### Checks on the Meaning and Effectiveness of the Treatment

Sometimes the independent variable that the researcher cares about is something that cannot be manipulated directly, often an internal state such as anxiety, and the researcher comes up with a treatment that is designed to produce that state in the participants such as threatening them with painful electric shocks, showing them scary films, or making them prepare a speech to deliver to a critical audience. The researchers’ *conceptual* variable here is anxiety. The hypothesis may involve the effects of anxiety on affiliation, or depth of processing, or creativity, but these hypotheses cannot be tested directly because we cannot directly create the changes in the brain that correspond to a feeling of anxiety. So we make a guess about the kind of situation that we think would produce anxiety in most people who are like our participants, and we put our participants in that situation.

But we might be wrong. The participants might think our terrifying film is just silly, or our infuriating confederate is not enraging, but just contemptible. So we add a direct measure of the state we wanted to create, often just by asking the participants to rate how fearful or angry they were, how credible they thought the communicator was, how much they liked the confederate, or whatever other internal state we were trying to create. In this way we seek empirical verification that the manipulation had the intended psychological effect.

We could also be wrong in other ways. Our heart-rending film may make our participants sad as we intended, but it may also simultaneously make them hostile toward the experimenter for showing it. Manipulation checks can also be used to rule out the possibility that the manipulation had unintended effects on other states. In order to show that sadness (and not any other variables) drives the effects, manipulation checks are used to show that a treatment designed to produce sadness did *not* produce hostility or other mental states that could plausibly interfere with the conclusions.

Manipulation checks have also been used to assess the effectiveness of a treatment. In Schachter’s (1959) work on affiliation, for example, he attempted to create anxiety by having a stern-looking experimenter in a white lab coat tell the participants that the experiment required them to undergo a series of shocks. Immediately afterwards, the experimenter asked the participants to fill out a scale designed to measure their anxiety in order to check on the effectiveness of the manipulation. Finally, they completed scales measuring their desire to wait for the experiment alone or with other participants (the key dependent variable, a measure of affiliation).

The anxiety manipulation was effective in the first experiment, but less so in the follow-ups where it did not produce significant increases in anxiety overall, though it may have for some participants. In some of these studies, Schachter used the participants’ anxiety ratings to reclassify them into high- and low-anxious groups: “in order to provide further insight into these data it seemed advisable to undertake an internal analysis; that is, to compare the responses of the truly anxious subjects with those of relatively calm subjects. If …there really is no relationship between anxiety and the affiliative tendency there should be no difference between these two groups of subjects” (1959, p 34). But there was a difference – a relationship that was non-significant when the randomly-assigned anxiety conditions were compared became significant when the participants were reassigned on the basis of their self-rated anxiety.

Manipulation checks provide opportunities for these internal analyses when treatments fail. In addition to checking on the effectiveness of the manipulation, they allow the researcher a second, correlational, method of checking on the plausibility of the hypothesis, even when the manipulation was ineffective. The idea of internal analysis has resurfaced in the modern methods literature where new approaches to account for the actual effects of the manipulation or treatment have been proposed (see Angrist and Pischke, 2009, for various implementations). This allows for the possibility that a participant assigned to a given condition may not experience the intended effect. The logic behind IMCs is somewhat similar, in that only data from the participants who were careful enough to actually read the questions (and presumably the text containing the treatment) are analyzed.

### Checks on Mediating Processes

Another, somewhat more recent, use of manipulation checks is to identify a mediating variable between the independent variable and the outcome. The manipulation is supposed to cause some internal state, but it is the internal state that is supposed to cause the outcome, and a statistical analysis of this second causal relation requires that the internal state be measured.

Mediation analysis is a means of demonstrating an intervening process through which an independent variable influences a dependent variable (Baron and Kenny, 1986). If you hypothesize that attractive people are more persuasive than unattractive people, you can manipulate attractiveness and measure persuasion. If you hypothesize that the *reason* that attractiveness increases persuasiveness is because attractive people seem more intelligent, you can also measure participants’ perceptions of the persuader’s intelligence. Intelligence then becomes a mediating variable. If attractiveness predicts perceived intelligence and perceived intelligence predicts persuasion, but attractiveness no longer predicts persuasion when you control for perceived intelligence, you can conclude that one reason that attractiveness makes people more persuasive is that it makes them seem more intelligent.

In a recent article, Lench et al. (2014) argue that manipulation checks are an important means of ruling out alternative hypotheses and establishing the causal role of the intended mental state in producing the outcome, and that the proper analysis of manipulation checks is the same as the analysis of hypothesized mediating variables: the researcher must show that the manipulation predicts the dependent variable and the responses to the manipulation check, and must also show that the responses to the manipulation check predict the dependent variable. They argue that if you believe that your independent variable treatment produces a mental state that in turn produces an outcome, obviously it is better to show that the manipulation check does produce the outcome than to simply show that your treatment produces both the response to the manipulation check and the final outcome. For these reasons, some suggest using manipulation checks within mediation analyses.

A manipulation check is not the same as the measure of a mediating variable, although the line can be blurry, as when the manipulation check is designed to measure whether the manipulation produced the intended psychological state. As we explain below, however, our general argument applies to *any* noticeable measure inserted into a study, whether it be a manipulation check or a measurement of a mediating variable.

## So What is the Problem?

### Psychological Problems

The problem is that the participant is a human being – not a solution in a Petrie dish or a crash dummy or even a plant. As researchers, we want the participant to be paying attention to the events we have arranged, noticing the manipulation and responding to it, and taking the measures seriously. We want the sequence of events to capture the participant’s attention and to unfold naturally, and the measures to seem natural and appropriate in the context of the participant’s experience. We want the participant to respond spontaneously, not to step outside the experimental context and wonder why the experimenter decided to include some particular task or question (Ellsworth and Gonzalez, 2003; Aronson, 2010; Ellsworth, 2010). But our participants are sentient, thinking, self-conscious human beings, trying to make sense of the events that they encounter and reacting to them as events. *We* may think of our manipulation check as a measure, but in many cases it is not simply a measure, but also a new event for the participant.

We want the participant to attend closely to the manipulation, but not to the manipulation check, but what we call a manipulation check could also be a manipulation. The participant may be watching someone suffer, or absorbed in making smart choices in a competitive game, or trying to come up with a presentation that won’t sound dumb in the Trier Social Stress test, and then suddenly the flow of events is interrupted. The experimenter now wants some ratings of compassion or hostility or anxiety, or asks the participant to spit into a test tube. These questions or saliva samples are not just measures, but events that happen to the participants, and responses to the dependent variable we care about can be changed by the experience of responding to a manipulation check. There is no reason to believe that a person who watches another person suffering is psychologically the same as a person who watches another person suffering and then fills out a bunch of scales designed to measure compassion. For example, the focus of attention may change from a concern with the other person to a concern with oneself. There is no reason to believe that a person who is caught up in trying to win is psychologically the same as a person who was trying to win but now answers a series of questions designed to measure hostility. Again, the focus of attention has changed, and now the person may have to reconsider the motives for competition. Cortisol levels may be elevated by the anticipation of the disgusting experience of spitting in public. A manipulation check may be able to tell us what the participant was feeling or thinking right *before* the manipulation check, provided that the participant is able and willing to tell us. It is quite possible that it can tell us nothing at all about what the participant was feeling or thinking right *after* the manipulation check, and those are the feelings and thoughts that we think will predict the dependent variable.

A manipulation check may affect the participant’s thinking in various ways. The most often mentioned concern (Parrot and Hertel, 1999) is that it tells the participant something about the researcher’s hypothesis. A set of questions about emotions or self-esteem or prejudice or liking for another person in the experiment suggests that the experimenter is interested in emotions or self-esteem or prejudice or liking. The participant may wonder why the experimenter cares, and may think about what happened right before the manipulation check (the independent variable) and whether that event was supposed to affect the answers to the questions.

But guessing the hypothesis is not the only danger of using manipulation check measures. In any given setting, answering the manipulation check questions may affect the participant’s thought processes in ways that are particular to that setting and thus particularly hard to identify. Filling out a set of scales in which you can express your dislike by criticizing the offensive person may serve as a way of expressing your aggression, so that by the time you get to the dependent variable you are feeling less aggressive than if you hadn’t had a chance to express your feelings. Keltner et al. (1993) found that sorrow decreased people’s perceptions of their general life satisfaction. But this effect disappeared if the participants were asked about their feelings after the induction of sorrow. Keltner et al. (1993) argue that describing one’s feelings after the treatment made people connect their feelings to the induction and therefore not connect them to their general life satisfaction.

On the other hand, filling out hostility scales may crystallize your vague feelings of hostility and make you feel even more angry (Kidd, 1976, citing Mallick and McCandless, 1966). Kühnen (2010) found that across a variety of paradigms fluency effects only occurred when participants were asked to rate how easy or difficult the recall task was (fluency) before they responded to the dependent variable measure, suggesting that the real independent variable was not just the easy or difficult recall task, but the conscious labeling of that task as easy or difficult required by the manipulation check. Schwarz has documented a dizzying array of effects that one question can have on the next question in surveys (e.g., Schwarz and Strack, 1991), and a verbal manipulation check is just like any other question. Like a survey question, it can influence what comes next.

Asking participants to describe their emotions can also change their physiological and brain responses to the emotional stimuli. Kassam and Mendes (2013) found that participants who were asked to rate how angry they were had qualitatively different cardiovascular responses to the anger manipulation than participants who were not, and Lieberman et al. (2007) found that asking participants to name the emotion in pictures of emotional facial expressions reduced the response of the amygdala to the faces (Creswell et al., 2007; Lieberman et al., 2007).

There are many possible ways in which a manipulation check might affect the participant. It could undo the effects of the manipulation; it could enhance the effects of the manipulation; or it could interact with the manipulation. There is no way to know which of these processes may be occurring in any particular experiment without empirically investigating it, but in any case the assumption that the manipulation check is a neutral unobtrusive measure of the effects of the manipulation may be unjustified.

Consider the implications for mediation analysis. The assumption is that A (the manipulation) leads to B (the assumed mediator, typically an internal state) which in turn leads to C (the dependent variable outcome). But if the measurement of B produces X (some effect of the measurement itself), then the logic fails. A may lead to B, but we can no longer conclude that B leads to C. X (the measurement effect) or an interaction of A and X or an interaction of B and X may lead to C. Would C have shown the same effects if B had not been measured? We cannot know unless we run the same experiment without measuring B and find that the A–C relationship is the same. Thus, if a researcher wants to go the route of using manipulation check measures, then the analysis and experimental design decisions are more complicated than merely conducting a simple mediation analysis.

If B is an IMC or attention check designed to identify participants who are not paying attention, the problem is the same. The participants who survive, who read the IMC carefully and answer correctly, may not be in the same mental state as those who were influenced by the independent variable but were not given an IMC. They may be extra wary, they may be annoyed at the experimenter for trying to trick them, they may be distracted by this peculiar question coming in the middle of a series of otherwise coherent and sensible questions, but we cannot assume that they are like participants who were never asked about sports and then told that the question was not really about sports. Hauser and Schwarz (2015) found that participants who first passed a common IMC spent more time on subsequent measures of analytic thinking and responded more reflectively than participants who completed the analytic thinking task before the IMC. They argue that IMCs tell participants that there is more to questions than meets the eye, and lead to increased deliberative thinking on reasoning tasks that follow IMCs.

Oppenheimer and his colleagues actually discussed these possibilities in their 2009 article, and their second study even demonstrates how IMCs can be used as interventions to increase participant attentiveness. However, while they cautioned that IMCs may undermine data quality due to participant reactance, they failed to consider that the intervening effects of IMCs may threaten the validity of research conclusions, and subsequent research on IMCs has not yet addressed this problem.

These considerations have implications for methods in reproducibility research. Preregistered direct replication projects emphasize the importance of including manipulation checks (Simons and Holcombe, 2014), but their inclusion in replications could create new problems. Checks on a manipulation’s effectiveness seem necessary for ensuring that a study’s original manipulation still produces the intended psychological state in a direct replication project (Schwarz and Strack, 2014). However, if manipulation checks or any other new measures are added to the procedure of a study that did not originally include them, the new study is no longer a “direct” replication. Because these questions can change people’s subsequent responses, including them in a replication limits a replicator’s ability to make claims about the reproducibility of the original study because the replicator has effectively run a different study.

These issues have been raised in discussions of registered replications. A multi-lab preregistered replication of the famous “pen in mouth” study (Strack et al., 1988) failed to replicate the original effect that a pen held between the teeth (inducing a smile) causes participants to rate cartoons as being funnier than a pen held between lips (Wagenmakers et al., 2016). However, the replication project added a manipulation check to the original design. Unlike the original study, the replication also focused a video camera on the participants and informed them that researchers would be recording their actions in order to verify that they were following instructions correctly (steps 16 and 17 in protocol; Beek et al., 2015). Strack (2016) suggested that pointing a video camera at participants may have induced a focus on the self that could interfere with misattributing amusement to the cartoons. Lengthy arguments about the validity of the replication project pervaded discussions in Psychological Methods Facebook groups and academic Twitter. Recently, Noah et al. (2018) conducted another pre-registered replication of the facial feedback study, this time manipulating the presence vs. absence of the video camera. The facial feedback effect replicated with the camera absent, but did not replicate with the camera present. However, the interaction of camera × facial feedback was not conventionally significant, so many still debate whether the Wagenmakers et al. (2016) non-replication was due to the interference of the manipulation check or due to a genuine failure to replicate the original effect. However, one thing is certain: adding a manipulation check to a replication project that was not present in the original study can cause others to question the relevance of the replication’s results to the reproducibility of the original effect.

### Analytical Problems

There are several ways in which manipulation checks and their use in analyses can be misleading on statistical grounds. One is that even if an experiment is conducted with random assignment, the use of a manipulation check in a mediation analysis still provides only correlational evidence of the hypothesized process, as in Schachter’s (1959) internal analyses. An experimental manipulation may influence the manipulation check and it may influence the final outcome, but the relation between the manipulation check and the outcome variable in this case is correlational. A mediation analysis cannot lead to causal interpretation unless there is an additional experimental manipulation designed to assess the association between the manipulation check used as a measure of the hypothesized internal mechanism and the outcome variable. Modern scholarship in causal inference shows more clearly the assumptions that need to be in place for a causal interpretation from a mediation analysis (Imai et al., 2011; Pirlott and MacKinnon, 2016). Several authors have argued that mediation analyses are often used when other methods of understanding psychological processes would be more effective (Spencer et al., 2005) and are often misused when crucial theoretical assumptions are not met (MacKinnon et al., 2007; Kerr, 2014).

A second issue is measurement error. Like all observed variables, manipulation checks include measurement error. Although the impact of measurement error on mediation analysis was pointed out in the original Baron and Kenny article, the literature has given little attention to this important point. We know that measurement error in a predictor used in a multiple regression can introduce bias and reduce statistical power (see Culpepper and Aguinis, 2011). However, it is not well known or appreciated that measurement error can change the sign of a regression slope (Cohen and Cohen, 1984). Cohen and Cohen provide examples of how moderate reliability (0.70) in one regression variable can change the sign of a regression weight; for example, measurement error can change a significantly negative slope into a significantly positive slope. We do not yet understand the role measurement error plays in more modern and sophisticated tests of the standard “a times b” paths in meditational analyses. There is much research to do in studying different methods of incorporating a manipulation check measure into the analysis, including, for example, comparing different approaches to handling a manipulation check measure, such as an instrumental variables approach and its related errors-in-predictor approach, a propensity score approach, the standard mediation framework, and other approaches (Morgan and Winship, 2014; VanderWeele, 2015).

A third issue is that the simplicity of the traditional mediation model may not sufficiently capture the complexity of the underlying relationship between manipulation, manipulation check and outcome variable. As discussed earlier, there could be complex relationships where, for example, the manipulation check could interact with the manipulation in its effect on the outcome variable. While the mediation framework can be extended to moderated mediation and mediated moderation, there are other approaches to modeling these more complex relationships involving extensions of the techniques mentioned in the previous paragraph.

What is clear is that more methodological research is needed to understand how to incorporate manipulation check measures into one’s analysis. There are promising alternatives currently available. The mediation framework may not be the best approach and in some cases may lead to incorrect conclusions.

## Solutions

### Manipulation Checks that are Not Manipulations

There are several questions to ask about a manipulation check in order to decide whether it is likely to be a manipulation.

Is it an event? Is it something new that happens to the participant? A questionnaire, or even a question, is an event (Schwarz, 1999). When something new happens, the participant may wonder, “What’s this about?” Almost all verbal measures are events, even essays that will later be coded for something the participant doesn’t know about. The participant may still ask, “What’s this about?” and it is a good idea for the experimenter to have an answer to this question. In the context of a cover story about creativity, an essay can be explained as measuring some type of creativity. If the cover story is about memory, the essay can be presented as a filler task, in order to let more time go by before the memory test. A good cover story makes sense of all parts of the study. If an event makes sense as part of the general framework of the experiment, if the participant has an answer to the question “What’s this about?” it is less likely to stand out as something the participant has to try to understand.

Non-verbal measures can also be events, and if they are, they are worth worrying about in the same way as verbal measures. If energy depletion is measured by asking the participant to squeeze an apparatus measuring grip strength, the participant may wonder about it. If stress is measured by having the participant spit into a tube several times during the experiment, this may be the feature of the experiment that stands out most to the participant, who may be disgusted or stressed by the procedure. A continuous physiological or video recording is not an event in the same way, because it exists both before and after the independent variable treatment (unless the researcher calls attention to it at a particular point by turning it on, fiddling with it, etc.). It may make the general setting appear unusual, and raise questions about generalizability, but the part of the stream of behavior that the experimenter later selects as a manipulation check is not an event that is noticeable to the participant.

Can the participant adjust her response to fit what she thinks is the hypothesis, undermine what she thinks is the hypothesis, or otherwise respond to information she has gotten from the manipulation check? Decades of research have demonstrated that “there is no necessary correspondence between what the subjects feel, believe, expect, or do, and what they say” (Carlsmith et al., 1976, p. 193; Kagan, 2007; Schwarz, 2007). In answering questions, people make inferences about the intentions of the questioner, interpreting each question in light of what has gone before, and adjust their answers to fit their perceptions of the intended meaning and purpose of the question. So a participant who is not scared may rate herself as scared because she perceives that the film she just saw was supposed to be frightening. Or a participant who is scared may not want to admit it.

Are there ways to check on the effectiveness of the manipulation without running the risk of influencing the participant’s response to the dependent variable measure? One common method is not to check on the manipulation until after the dependent variable has already been measured. This guarantees that the response to the dependent variable was not affected by the manipulation check. However, moving the manipulation check to the end of the study may compromise its validity as a manipulation check. Participants may not remember what they were feeling and thinking before the dependent variable measure was taken or they may be unwilling to admit that they were scared or angry. Or worse, just as in the usual sequence the dependent variable could be affected by both the manipulation and the manipulation check, so to, in this revised sequence, the manipulation check could be affected by both the manipulation and the measurement of the dependent variable. If the participant has successfully resolved dissonance by changing her attitude, then she may not report any dissonance, or if she has successfully punished her enemy, she may not report any hostility. People are notoriously unaware of changes in their attitudes or feelings, and tend to believe that what they are feeling now is what they have felt all along (Bem and McConnell, 1970). It would be wrong to conclude that if the post-dependent variable manipulation check showed no effects, the treatment failed to create the predicted mental state. The mental state might have been there when it was supposed to be, but disappeared by the time it was measured. Likewise, a “successful” manipulation check is no guarantee that the treatment produced the intended state. The person may infer their state not by remembering what they felt at the time of the treatment, but by remembering how they responded to the dependent variable measure. For example, if they punished the confederate, they may infer that they must have been angry.

Postponing IMCs until the end of the study is also problematic: as long studies wear on, participants often pay less attention and resort to satisficing (Krosnick, 1999; Galesic and Bosnjak, 2009), so they may fail IMCs at the end of the study even if they had been attentive earlier. This would make end-of-study IMCs overly-conservative measures of attention. Additionally, attentiveness ebbs and flows throughout the course of an experiment. IMC’s can only measure attention at the moment of administration, so attentiveness during the crucial task may be different from attentiveness during an IMC that occurs later. Berinsky et al. (2014) asked participants four IMC questions spaced out over the course of a survey and found that passing one IMC correlated with passing another between *r* = 0.38 and *r* = 0.46. It is unrealistic to assume that attention remains constant throughout a study or that a single IMC is a reliable measure of attention. Participants may be inattentive during a crucial measure but pass a postponed IMC or vice versa.

There are many ways to increase attentiveness besides using IMCs. The design of the survey should be considered; shorter surveys, engaging surveys, and surveys administered in person hold attention well (Krosnick, 1999; Galesic and Bosnjak, 2009; Oppenheimer et al., 2009). Most use of IMCs is online on survey platforms such as Amazon’s Mechanical Turk, where researchers are especially concerned about inattentive participants. However, research shows that this concern may be misplaced. MTurkers have been incentivized to be equally or more attentive than college subject pool students (Hauser and Schwarz, 2016), especially MTurkers who meet certain worker restrictions that are easy to implement (Peer et al., 2014).

The only way to find out whether a manipulation check affected the outcome of a study is by an experiment: run the study with and without the manipulation check. If the results are the same, we can conclude that the manipulation check did not interfere with the process we are studying. The logic is the same as the logic of checking for pretest sensitization: If we ask participants about their attitudes toward an outgroup right before we show them our anti-prejudice film, we cannot tell whether the effects are due to the film (as we hypothesize), to the pretest, or to the combination of the pretest and the film. Campbell and Stanley (1963) recommended the Solomon four-group design to deal with this problem. If the experiment involves two conditions – *pretest-treatment-posttest* and *pretest-no treatment-posttest*, then two more conditions must be added to discover or rule out the effects of the pretest – a *no pretest-treatment-posttest* condition and a *no pretest-no treatment-posttest* condition. In the case of manipulation checks, the four conditions would be *treatment-manipulation check-dependent variable measure, no treatment-manipulation check-dependent variable measure, treatment-no manipulation check-dependent variable measure*, and *no treatment-no manipulation check-dependent variable measure*, or systematically varying the placement of the manipulation check to come before or after the measurement of the dependent variable. However, this design is extremely rare (see **Table 1**).

Another method is to find a manipulation check that is not an event that the participant can notice. Webb et al. (1966) recommended the use of *unobtrusive measures*, measures that could be taken without the participant’s awareness. Examples include some observational measures, some behavioral measures, some physiological measures, and even some analyses of verbal measures.

Observational measures, common in animal behavior and developmental psychology, involve an observer recording the behavior of an individual or a group, either directly, or usually less obtrusively, on a videotape. Do the participants in the anger condition frown more? Or do observers, blind to condition, rate these people as angrier, more confident, or more certain than people in the other conditions? If the video camera in the registered replication of the Strack pen-in-mouth study had been concealed from the participants, rather than explicitly brought to their attention, it would have likely been unobtrusive. Of course this sort of manipulation check can typically only be used when the participant is doing something other than filling out forms or sitting at a computer.

Sometimes aspects of a behavior can be measured without the need for human observers. Reaction time, used to measure speed of mental processing, is the most common example. Although typically used as a dependent variable measure, there is no reason that reaction time could not be used as a manipulation check, for example if the researcher wanted to vary whether problems were easy or difficult, or stimuli were clear or ambiguous. Variability of responses can also be used by researchers as a check on how closely the participants are paying attention. If the participant “straight lines” by checking the same number on all of the rating scales, or agrees with both direct and reverse-coded items, then the researcher can conclude that the participant was not paying attention to the manipulation (for a review of these techniques, see Curran, 2016).

Other aspects of behavior besides speed and variability are potentially measurable. For example, researchers have examined chosen distance from a confederate or speech errors as measures of discomfort (e.g., Word et al., 1974). Physiological measures such as heart rate, blood pressure, GSR, pupil dilation, or respiration rate are often used as measures of stress or arousal; facial expressions as measures of particular emotions; and measures of brain activity are increasingly capable of measuring specific mental processes (Cacioppo et al., 2017).

However, not all behavioral are unobtrusive. We generally assume that physiological and brain processes cannot be consciously controlled, but behavioral responses might be. If the participant has just completed a difficult physical task and then is given a measure of grip strength, she may squeeze less hard because she knows she’s supposed to be feeling weak. Or if she has been told that the person she is about to talk to is warm and similar to herself (e.g., as a manipulation of liking) she may smile a lot because she thinks that’s expected of her. While measuring smiling is likely to be a much better manipulation check than asking “How likeable is this person?” because the question about liking is a salient event and the unobtrusive measure of smiles is not, smiling is not completely beyond conscious control.

Even verbal measures can sometimes be used unobtrusively. A researcher interested in creating a sense of independence or interdependence can ask the participant to write an open-ended account of some experience and then code how often the participant uses “we” instead of “I.” A researcher interested in arousing positive vs. negative emotions or fear vs. anger can code the frequency of positive vs. negative or fearful vs. angry words. The Linguistic Inquiry and Word Count (LIWC; Pennebaker et al., 2001) allows for measurement of a large number of linguistic variables, or the researcher can devise a coding scheme specifically tailored to the variable he or she is trying to manipulate (Dehghani et al., 2017).

Although these measures are verbal, they are likely to be far less obtrusive than the usual rating scales used as manipulation checks. Writing a brief essay about one’s feelings or some experience is not the same as responding to a scale that says “How anxious (or hostile, or interdependent, or compassionate) do you feel right now?” These direct measures communicate that the experimenter is interested in anxious (or hostile, or interdependent, or compassionate) feelings. The participant generally has no idea what the experimenter will be looking for in coding the essay, or even whether it will be coded at all. A cover story in which writing an essay makes sense in terms of the participant’s understanding of the purpose of the experiment reduces the probability that she will stop and wonder why the experimenter is giving her this task.

Continuous unconscious online measures that begin before the independent variables are introduced and continue until the end of the study may be safe. The experimenter looks for changes that occur from before to after the independent variable manipulation in brain or autonomic nervous system activity, in the rate of speech hesitations, in the number of times the participant looks away from the stimulus, etc., and changes that occur in the experimental group but not in the control group are fairly trustworthy manipulation checks. There may be some costs in terms of generalizability or “ecological validity” if the measuring apparatus itself is obtrusive, such as a polygraph or an FMRI scanner, but these are problems of external rather than internal validity. The experimenter hopes that the scanner or the videotape camera or any other measuring device will become a constant background factor of the experiment, so that the participant soon stops paying attention to it and responds only to the experimental events. The experimenter also hopes that there are no complicated interactions between the presence of the manipulation check device and the key dependent variables.

A common objection to the use of behavioral measures, either as dependent variables or as manipulation checks, is that they have multiple meanings. Keeping one’s distance could mean shyness, not hostility; interrupting could mean fear of exclusion, not dominance, and so on. Many behaviors do not have one-to-one correspondence with underlying states, and it is important for the researcher to consider other possible meanings of the behavior and design the experimental context so that the intended meaning is the one that is plausible in that context (see Ellsworth and Gonzalez, 2003, for a discussion of this issue). Researchers have to make assumptions about the meaning of the behavior in using these measures. But the multiple meanings of *verbal* measures, and their sensitivity to small contextual factors have been known for decades: there is no reason to believe that a rating scale provides a pure and unambiguous measure of the mental states corresponding to the words on the scale, and there are dozens of well-documented reasons to believe that it does not (Schwarz, 1999; Kagan, 2007). Filling out a scale is an event that interrupts the participant’s experience, and the response to the scale is not just a reflection of the participant’s experience before the scale appeared, but also a response to what the participant thinks about the scale itself, and this response may also change the experience so that even if the experimenter succeeded in creating a particular mental state, it may no longer exist after the measurement.

### A Better Solution: Checking the Manipulation in Pilot Research

Finally, one can conduct the manipulation check before actually running the experiment, in a pilot study whose whole purpose is to find out whether the treatment successfully produces the intended psychological state. The researcher creates or selects events or films or vignettes or some other stimuli designed to create different states – contempt and compassion, joy and relief, high credibility and low credibility and so on, presents them to the participants, and then asks what they meant to the participants (i.e., administers the manipulation check). Sometimes the treatments produce clear differences in the participants’ state in the way that was expected. Sometimes there are no significant differences – both treatments produce similar states, or there is so much variability in responses to one or both treatments that the noise drowns out the signal, or a treatment means something quite different from what the researcher thought it would. This is disappointing and frustrating and means that we have to try again, guided by our mistakes. It takes time, but in the long run it is better to find out that your treatments are ineffective and to come up with better ones before running the whole experiment. A manipulation check within the context of the real experiment may be a less trustworthy method of discovering the mistake, for all the reasons we have described.

If the researcher has shown through a pilot study that the independent variable produces the expected belief, emotion, or other state in people like the participants to be run in the actual experiment, then there is no need to clutter up the actual experiment with an intrusive measure that may disrupt the flow of events and have an independent or interactive effect on the dependent variable measures that the researcher cares about. This is the same logic that underlies Spencer et al.’s (2005) recommendation for the use of separate experiments instead of mediational analyses. This approach essentially establishes the validity of the manipulation (i.e., that it produces the desired effect) on a different group of participants. Of course, the pilot test should be run on people like the actual participants pretty soon before the actual study is run, in order to be confident that the participants in the real experiment will respond in the same way. Using materials that were “validated” on a sample 20 years ago is no guarantee that present-day participants will see them in the same way – fashions change, familiar stimuli become unfamiliar and unfamiliar stimuli (e.g., exciting new technologies) may become familiar or even passé. Even words change in their frequency and their meanings over time (Ramscar, 2016). The purpose of pilot testing is to discover whether a treatment is effective for a particular group of participants at a particular time – people like the people who will be in the actual experiment. The strategy of testing the meaning of a manipulation in a pilot study is also useful for researchers attempting to conduct a direct replication, as it allows them to check on the effectiveness of the manipulation for the new sample while leaving the replication itself intact.

## Conclusion

Validating the meaning of manipulations is important, and we are not advocating that manipulation checks be abandoned. We are arguing against the mindless inclusion of obtrusive measures – manipulation checks, measures of mediating variables, or any other measures between the manipulation and the dependent variable measure – that may influence the thoughts and behaviors of participants. The addition of a manipulation check in the service of testing validity can introduce new problems that threaten validity. By adding additional measures the researcher may change the internal psychological processes. There is more than one way that a manipulation can be validated, and researchers should give the same careful consideration to their choice of a manipulation check as they do to their choice of dependent variable measures. Authors should justify including a manipulation check with an experiment if they chose to do so, explaining why it is necessary and why it is unlikely to affect their conclusions. Often the best choice may be to forego including a manipulation check in the actual study by establishing the effectiveness of the manipulation through other means such as in pilot work. Editors and reviewers should evaluate whether a particular manipulation check improves or impairs the quality of any given study rather than assuming that using a manipulation check automatically improves it.

## Author Contributions

PE wrote the initial draft of the manuscript, upon which DH and RG provided critical revisions.

## Conflict of Interest Statement

The authors declare that the research was conducted in the absence of any commercial or financial relationships that could be construed as a potential conflict of interest.
